# Kodierung von Kindesmisshandlung und -vernachlässigung in Kliniken in Deutschland: Übersicht und Einordnung der Datenlage im Hellfeld

**DOI:** 10.1007/s00103-024-04002-4

**Published:** 2025-01-14

**Authors:** Teresa Walter, Jörg M. Fegert, Andreas Jud

**Affiliations:** 1 Klinik für Kinder- und Jugendpsychiatrie, Psychosomatik und Psychotherapie, Universitätsklinikum Ulm, Steinhövelstraße 3, 89075 Ulm, Deutschland; 2 Kompetenzzentrum Kinderschutz in der Medizin Baden-Württemberg (com.can; Competence Center Child Abuse and Neglect), Ulm, Deutschland; 3 Kompetenzbereich Prävention Psychische Gesundheit im Kompetenznetzwerk Präventivmedizin Baden-Württemberg, Ulm, Deutschland

**Keywords:** Kindesmissbrauch, Administrative Daten, T74, ICD-10, OPS, Child abuse, Administrative data, T74, ICD-10, OPS

## Abstract

**Hintergrund:**

Kindesmisshandlung ist in Deutschland ein gravierendes Problem. Um entsprechend effektive Präventionsmaßnahmen zu planen, stellen insbesondere erhobene Krankenhausdaten zu Kindesmisshandlung eine wichtige Datengrundlage dar. Damit kann beurteilt werden, inwieweit gefährdete Kinder und Jugendliche identifiziert, unterstützt und geschützt werden. Eine systematische Auswertung und Einordnung dieser Krankenhausdaten in Deutschland fehlt bislang. Ziel der Arbeit war es deshalb, einen Überblick über die Datenlage in diesem Hellfeld zu erlangen und damit die bestehende Forschungslücke zu schließen.

**Methode:**

Deskriptive Auswertung (teil-)stationärer Daten zu Kindesmisshandlung und -vernachlässigung (Nebendiagnose in der Internationalen statistischen Klassifikation der Krankheiten und verwandter Gesundheitsprobleme, 10. Revision (ICD-10): T74.0, T74.1, T74.2, T74.3, T74.8, T74.9 + Prozedur OPS: 1‑945.0, 1‑945.1) aus dem Erhebungszeitraum 2019 bis 2023. Betrachtet werden jeweils die Variablen Alter, Geschlecht sowie misshandlungsrelevante Hauptdiagnosen. Der Zugang zu den Daten erfolgte über den Datenbrowser des Instituts für das Entgeltsystem im Krankenhaus (InEK).

**Ergebnisse:**

Die verschwindend geringe Anzahl diagnostizierter und dokumentierter Fälle von Kindesmisshandlung im (teil-)stationären klinischen Setting steht in starkem Kontrast zu den Dunkelfeldprävalenzen bevölkerungsrepräsentativer Untersuchungen. Eine erhebliche Untererfassung aller Misshandlungsformen wird deutlich, die verschiedenen Formen bei Jungen und Mädchen unterschiedlich häufig erkannt. Darüber hinaus wird die oft fehlende Verknüpfung kindesmisshandlungsrelevanter Diagnosen und geleisteter Prozeduren ersichtlich.

**Schlussfolgerung:**

Neben Sensibilisierung sowie Aus‑, Fort- und Weiterbildung von Fachkräften im Gesundheitswesen besteht die Notwendigkeit von gezielten politischen Veränderungen.

## Abstract (en)

**Background:**

Child maltreatment is a significant problem in Germany. Hospital data on child maltreatment serve as a crucial foundation for planning effective prevention measures. These data enable an assessment of the extent to which at-risk children and adolescents are identified, supported, and protected. A systematic evaluation and classification of all hospital data relevant to child maltreatment in Germany is still lacking. Therefore, the aim of this article is to gain an overview of the data in this field and thus bridge the gap.

**Methods:**

Descriptive analyses of (partially) inpatient data on child abuse and neglect (secondary diagnosis in ICD-10: T74.0, T74.1, T74.2, T74.3, T74.8, T74.9) + (procedure OPS: 1‑945.0, 1‑945.1) from the 2019–2023 data collection period. The variables age, gender, and main diagnoses relevant to maltreatment are considered in each case. The data were analyzed via the publicly accessible data browser of the Institute for the Hospital Remuneration System (InEK).

**Results:**

The number of diagnosed cases of child maltreatment of (partially) inpatients is detached from prevalence rates in the population; in consequence, there is a significant underreporting of all forms of maltreatment, with boys and girls being recognized differently for different forms of maltreatment. In addition, the frequent lack of a link between child maltreatment-related diagnoses and procedures performed becomes apparent.

**Conclusion:**

In addition to raising awareness, training, and further education of healthcare professionals, there is also a need for investment in political change.

## Hintergrund

Kindesmisshandlung und -vernachlässigung stellen in Deutschland ein gravierendes Problem dar. Die aktuellste, repräsentative Prävalenzstudie in Deutschland von Witt et al. (2016) verdeutlicht das beträchtliche Ausmaß im Dunkelfeld: Von den 2504 befragten Personen berichteten 6,5 % über mindestens moderaten emotionalen, 6,6 % über körperlichen und 7,6 % über sexuellen Missbrauch. Des Weiteren berichteten 13,3 % von emotionaler und 22,4 % von physischer Vernachlässigung während ihrer Kindheit und Jugend [1]. Laut Kinder- und Jugendhilfestatistik meldeten Jugendämter in Deutschland im Jahr 2022 62.279 Fälle von Kindeswohlgefährdung. Bei 35 % gab es Hinweise auf psychische Misshandlung, bei 27 % wurde körperliche Misshandlung identifiziert und bei 5 % gab es Anzeichen für sexuellen Missbrauch [2].

Das Ausmaß von Kindesmisshandlung und -vernachlässigung verdeutlicht die Notwendigkeit entsprechender Präventionsmaßnahmen und Interventionen, um gefährdete Kinder und Jugendliche zu schützen, den negativen physischen, psychischen und sozialen Auswirkungen von Kindesmisshandlung [3] entgegenzuwirken und damit auch die enormen gesellschaftlichen Folgekosten [4] zu minimieren. Wenn es darum geht, effektive Interventionen und Präventionsmaßnahmen für gefährdete Kinder und Jugendliche zu planen, spielen Daten aus dem Hellfeld eine entscheidende Rolle, um beurteilen zu können, inwieweit Organisationen gefährdete Kinder identifizieren, unterstützen und schützen und inwieweit gefährdete Kinder gleichermaßen erkannt und betreut werden [5, 6]. Da Ärzt*innen Schlüsselpersonen für das Erkennen und das entsprechende Diagnostizieren von Anzeichen der Kindeswohlgefährdung sind [7], stellen insbesondere Krankenhausdaten zu Kindesmisshandlung basierend auf den Diagnosecodes der ICD-10-GM (Internationale statistische Klassifikation der Krankheiten und verwandter Gesundheitsprobleme, 10. Revision, German Modification) eine bedeutende Datengrundlage für nationale und vergleichende internationale Forschung dar [5, 8–10]. Sowohl im Rahmen des nationalen als auch des internationalen Fachdiskurses wird jedoch deutlich, dass im Gegensatz zur nationalen und internationalen Forschung im Dunkelfeld ein Mangel an zuverlässigen und validen administrativen Daten über die Häufigkeit von Kindesmisshandlung im Hellfeld besteht [10–12].

Wichtig ist deshalb eine Optimierung der Datenerfassung. Dafür ist es hilfreich, zunächst die bestehende Datenlage nachzuvollziehen und einzuordnen, um dann gezielte Maßnahmen zur Verbesserung der Datenerhebung und -qualität zu entwickeln.

Untersuchungen zu ICD-Diagnosecodes für Kindesmisshandlung im (teil-)stationären Krankenhaussetting aus den Jahren 2019 und 2020 in Deutschland fokussierten sich bislang auf eine detailliertere Betrachtung des Diagnosecodes zu sexuellem Missbrauch. Daraus wird ersichtlich, dass jährlich gerade einmal um die 130 Fälle von sexuellem Missbrauch kodiert wurden [11]. Eine weitere Studie untersuchte zwar in einem Kinderkrankenhaus die Erfassung von Kindesmissbrauch, sexuellem Missbrauch und Vernachlässigung bei stationären, teilstationären und ambulanten Fällen, jedoch wurden keine Diagnosecodes einbezogen [13]. Studien im internationalen Kontext haben sich ebenfalls mit Codes zu Kindesmisshandlung im klinischen stationären Krankenhaussetting befasst. Hier lag der Fokus insbesondere auf der Untersuchung der ICD-Codes zu körperlichem Missbrauch im Zusammenhang mit Frakturen. Von Interesse waren unter anderem die (Entwicklungen der) Inzidenzen der Hospitalisierungen [14–17].

Auf Grundlage vorhandener Studien zeigt sich, dass eine systematische Erfassung, Auswertung und Einordnung aller kindesmisshandlungsrelevanten Codes im Rahmen (teil-)stationärer Krankenhausaufenthalte in Deutschland bislang fehlen.

Ziel der vorliegenden Arbeit ist es deshalb, durch die Analyse von Daten zu Kindesmisshandlung in Kliniken und Krankenhäusern in Deutschland, die über den Datenbrowser des Instituts für das Entgeltsystem im Krankenhaus (InEK) abgerufen werden können, erstmals einen Überblick über die Datenlage in diesem Hellfeld zu erlangen und somit die bestehende Forschungslücke zu schließen. Die Darstellung der erhobenen Daten ermöglicht es, die bisherige Untererfassung von Misshandlung und Vernachlässigung in Kliniken in Deutschland systematisch einzuordnen. Zusätzlich sollen Ansatzpunkte für eine Optimierung der Datenerfassung aufgezeigt werden.

Konkret sollen dabei folgende Fragestellungen beantwortet werden:

1. Welche Daten zu Kindesmisshandlung und -vernachlässigung werden in Kliniken in Deutschland erfasst und wie häufig werden sie erhoben?
2. Inwieweit wird durch die Einordnung der Krankenhausdaten in bestehende Hell- und Dunkelfelddaten eine Untererfassung von Kindesmisshandlung und -vernachlässigung in Kliniken in Deutschland ersichtlich?
3. Welche potenziellen Ansatzpunkte für eine Verbesserung der Datenerfassung können mittels der erhobenen Daten identifiziert werden?

## Methoden

Die vorliegende Analyse erfolgte im Rahmen des EU-geförderten Projektes „Training To Improve Child Abuse and Neglect Diagnostic and Administrative Coding“ (TICANDAC)^1^. Das Projekt, eine deutsch-schwedische Kooperation, soll zur Prävention aller Formen von Gewalt gegen Kinder beitragen, indem es die nationale Datenerhebung unterstützt und verbessert.

Um einen Überblick über die bestehende Datenlage zur Dokumentation von Kindesmisshandlung im Hellfeld zu erlangen, erfolgten die Erfassung und Auswertung (teil-)stationärer Daten zur Kodierung in Kliniken und Krankenhäusern in Deutschland. Einsicht in die relevanten Daten und deren Analyse ermöglicht der öffentlich zugängliche Datenbrowser des Institutes für das Entgeltsystem im Krankenhaus (InEK). Gemäß § 21 Abs. 3b KHentG des 2. Bevölkerungsschutzgesetzes besteht für Krankenhäuser die Verpflichtung zur Datenlieferung an eben diese Datenstelle. Die Daten sollen „eine aussagekräftige und belastbare Informationsgrundlage schaffen“, wodurch Erkenntnisse aus weitergehenden Analysen der anonymisiert vorliegenden Daten unter anderem auch für die Wissenschaft von großem Interesse sind [18].

Durch Änderungen der Kodierrichtlinie DKR 1915 im Jahr 2013 ist die Kodierung von Kindesmisshandlung innerhalb des Fallpauschalensystems G‑DRG (German Diagnosis Related Groups) durch die ICD-Diagnosegruppe T74.x „Missbrauch von Personen“ möglich. Diese Diagnosereihe umfasst insgesamt 6 Codes, die verschiedene Misshandlungsformen einschließen. Formal betrachtet können die aufgeführten T74.x -Codes sowohl als Haupt- als auch als Nebendiagnose vergeben werden, da es sich um Primär-Codes handelt. In der Praxis ist die Vergabe als Hauptdiagnose allerdings unüblich, da zunächst die akute Verletzung – der Grund für die stationäre Aufnahme und hauptsächliche Behandlung – kodiert wird. Dieses Vorgehen wird auch in der offiziellen Code-Information nahegelegt [19].

Neben der Möglichkeit, Kindesmisshandlung und -vernachlässigung als Diagnose zu kodieren, kann seit 2013 auch die geleistete Prozedur der multiprofessionellen Abklärung bei Verdacht auf Kindeswohlgefährdung verschlüsselt werden. Hierzu wurden die Codes 1‑945.x in den Operationen- und Prozedurenschlüssel (OPS) integriert.

### Datenerhebung.

Aufgrund des Forschungsinteresses wurden ausschließlich (teil-)stationäre Fälle im Alter von 0–17 Jahren berücksichtigt. Erfasst wurden die Variablen Alter, Geschlecht, Haupt- und Nebendiagnosen sowie Prozeduren.

Es wurden folgende *ICD-10-Diagnosecodes als Nebendiagnose*^2^ erfasst:

- *T74.0* Imstichlassen oder Vernachlässigen,
- *T74.1* Körperlicher Missbrauch^3^,
- *T74.2* Sexueller Missbrauch,
- *T74.3* Psychischer Missbrauch,
- *T74.8* Sonstige Formen des Missbrauchs,
- *T74.9* Missbrauch von Personen, nicht näher bezeichnet.

Aus dem *Operationen- und Prozedurenschlüssel (OPS)* wurden die Daten für folgende Codes erhoben:

- *1‑945.0* Diagnostik bei Verdacht auf Gefährdung von Kindeswohl und Kindergesundheit – ohne weitere Maßnahme,
- *1‑945.1* Diagnostik bei Verdacht auf Gefährdung von Kindeswohl und Kindergesundheit – mit Durchführung von mindestens einer spezifisch protokollierten Fallkonferenz.

Da innerhalb des InEK-Datenbrowsers erst seit dem Datenjahr 2019 eine Auswahl an Selektionskriterien möglich ist, was eine differenzierte Darstellung relevanter statistischer Kennwerte ermöglicht, fokussiert sich der vorliegende Beitrag auf die Datenjahre 2019–2023. Die Jahre 2013–2018, die aufgrund der aufgezeigten Entwicklungen in den rechtlichen Regelungen für die Kodierung von Kindesmisshandlung grundsätzlich relevant wären, können bei der Auswertung nicht berücksichtigt werden, da hier nicht die vollständigen Fallzahlen zu den benötigten Diagnosen und Prozeduren ermittelt werden können (hier besteht keine Filtermöglichkeit nach einzelnen Diagnosen und Prozeduren, sondern es ist ausschließlich eine Gruppierung nach DRG möglich. Eine gezielte Selektion nach Geschlecht, Alter etc. für spezifische Diagnosen und Prozeduren ist ebenfalls nicht möglich). Um eine Rückführbarkeit von Daten auf einzelne Personen zu vermeiden, werden darüber hinaus innerhalb des InEK-Datenbrowsers Haupt- und Nebendiagnosen und Prozeduren erst ab 5 Fällen angezeigt. Ausprägungen von unter 5 Fällen werden unter der Bezeichnung „Rest“ aufsummiert dargestellt. Daten zur Anzahl der Fälle in Fachabteilungen sind nur von 2021–2023 verfügbar.

### Datenanalyse.

Die relevanten Daten wurden in Excel erfasst und dort mittels deskriptiver statistischer Analysen ausgewertet. Für die Jahre 2019–2023 erfolgte für die Diagnosegruppe T74.x und die Prozedurencodes 1‑945.x jeweils die Berechnung des (prozentualen) Mittels der Variablen Alter, Geschlecht, Fachabteilungen, Prozeduren sowie der vergebenen Hauptdiagnosen im Zusammenhang mit der Diagnosegruppe T74.x.

## Ergebnisse

### Stichprobe.

Die Anzahl (teil-)stationärer Fälle (0–17 Jahre) umfasst im Schnitt jährlich 1.893.794. 52,88 % der Fälle sind weiblich, 47,11 % männlich und bei 0,01 % der Fälle ist das Geschlecht unbekannt (Tab. 1).

**Tab. 1:** Stichprobenbeschreibung der Jahre 2019–2023 (Daten für Fachabteilungen waren erst ab 2021 verfügbar). Datenquelle: Institut für das Entgeltsystem im Krankenhaus (InEK)

	(Teil-)stationäre Fälle	Diagnosegruppe T74.x	Diagnosegruppe 1‑945.x
**⌀ Anzahl Fälle pro Jahr**	**1.893.794**	**1231**	**2192**
*⌀ Geschlecht in %*			
Weiblich	52,88 %	51,91 %	48,70 %
Männlich	47,11 %	48,08 %	51,29 %
Divers	0,00 %	0,01 %	0,01 %
Unbekannt	0,01 %	0,00 %	0,00 %
*⌀ Alter in %*			
< 1 Jahr	49,04 %	34,87 %	39,32 %
1–2 Jahre	9,79 %	14,32 %	14,04 %
3–5 Jahre	9,69 %	12,76 %	13,18 %
6–9 Jahre	8,71 %	10,50 %	12,00 %
10–15 Jahre	15,14 %	20,13 %	16,78 %
16–17 Jahre	7,27 %	7,42 %	4,68 %
*⌀ Anzahl Fälle Fachabteilungen pro Jahr (2021–2023)*			
Pädiatrie	–	1108	1646
Kinderchirurgie	–	129	297
Frauenheilkunde und Geburtshilfe	–	72	132
Unfallchirurgie	–	13	33
Allgemeine Chirurgie	–	5	7
Intensivmedizin	–	9	28
Neurochirurgie	–	5	10

*T74.x* „Missbrauch von Personen“ (Diagnosegruppe der Internationalen Klassifikation der Krankheiten – ICD-10), *1‑945.x* „Prozedur der Abklärung bei Verdacht auf Kindeswohlgefährdung“ (Diagnosegruppe des Operationen- und Prozedurenschlüssels – OPS)

Die Codes für Misshandlungsformen der Diagnosegruppe T74.x wurden im Schnitt 1231 Mal jährlich vergeben. 51,91 % der Fälle sind männlich, 48,08 % weiblich und 0,01 % divers. Die Kodierung der T74.x-Codes erfolgte im Schnitt am häufigsten in den Fachabteilungen der Pädiatrie (*n* = 1108), der Kinderchirurgie (*n* = 129) und der Frauenheilkunde und Geburtshilfe (*n* = 72).

Die Prozeduren 1‑945.x wurden jährlich im Schnitt 2192 Mal kodiert. 51,29 % der Fälle sind männlich, 48,70 % weiblich und 0,01 % divers. Die Kodierung der OPS-1-945.x-Codes erfolgte im Schnitt ebenfalls am häufigsten in den Fachabteilungen der Pädiatrie (*n* = 1646), der Kinderchirurgie (*n* = 297) und der Frauenheilkunde und Geburtshilfe (*n* = 132).

### Anzahl kodierter Fälle von Kindesmisshandlung und -vernachlässigung.

**Abb. 1:**
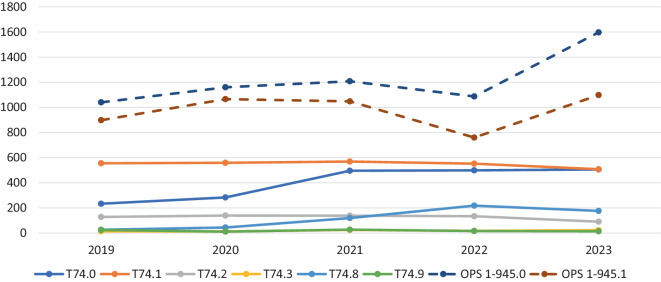
Entwicklung der Kodierung der Diagnosegruppen T74.x als Nebendiagnose und OPS 1‑945.x. (Datenquelle: Institut für das Entgeltsystem im Krankenhaus (InEK); eigene Abbildung) *T74.x* „Missbrauch von Personen“ (Diagnosegruppe der Internationalen Klassifikation der Krankheiten – ICD-10), *1‑945.x* „Prozedur der Abklärung bei Verdacht auf Kindeswohlgefährdung“ (Diagnosegruppe des Operationen- und Prozedurenschlüssels – OPS), *T74.0* Imstichlassen oder Vernachlässigen, *T74.1* Körperlicher Missbrauch, *T74.2* Sexueller Missbrauch, *T74.3* Psychischer Missbrauch, *T74.8* Sonstige Formen des Missbrauchs, *T74.9* Missbrauch von Personen, nicht näher bezeichnet, *1‑945.0* Diagnostik bei Verdacht auf Gefährdung von Kindeswohl und Kindergesundheit – ohne weitere Maßnahme, *1‑945.1* Diagnostik bei Verdacht auf Gefährdung von Kindeswohl und Kindergesundheit – mit Durchführung von mindestens einer spezifisch protokollierten Fallkonferenz

Abb. 1 zeigt die Anzahl der vergebenen Codes der Diagnosegruppe T74.x der ICD-10 sowie OPS 1‑945.x der vergangenen 5 Jahre und stellt deren Verlauf über die Jahre 2019–2023 dar. Aus den Fallzahlen ergibt sich folgende durchschnittliche jährliche Erfassung der einzelnen Formen von Kindesmisshandlung sowie ihr prozentualer Anteil an der Gesamterfassung von T74.x-Codes für Kindesmisshandlung: 403 Fälle (33 %) Vernachlässigung und Imstichlassen (T74.0), 549 Fälle (45 %) körperlicher Missbrauch (T74.1), 126 Fälle (10 %) sexueller Missbrauch (T74.2) und 18 Fälle (0,8 %) psychischer Missbrauch (T74.3). Insbesondere bei den T74.x-Codes zeigt sich nur eine geringfügige bis stagnierende Entwicklung in Bezug auf die Dokumentation. Ausnahme ist eine erkennbare Zunahme im Jahr 2021 um 213 Fälle bei der Kodierung von Imstichlassen/Vernachlässigung (T74.0). Ebenfalls ein deutlicher Anstieg ist bei der Vergabe der beiden Prozedurencodes 1‑945.0 und 1‑945.1 zu erkennen: Im Vergleich zum Vorjahr erfolgte 2023 eine Zunahme um 510 (1–945.0) und 338 Fälle (1–945.1).

### Häufigkeitsverteilungen nach Alter und Geschlecht.

**Abb. 2:**
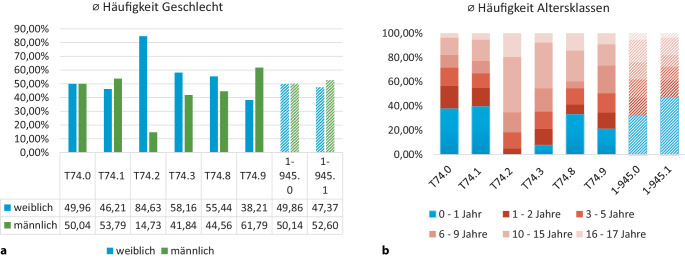
Geschlechterverteilung (**a**) und Altersverteilung (**b**) bei (Verdacht auf) Kindesmisshandlung. (Datenquelle: Institut für das Entgeltsystem im Krankenhaus (InEK)). *T74.0* Imstichlassen oder Vernachlässigen, *T74.1* Körperlicher Missbrauch, *T74.2* Sexueller Missbrauch, *T74.3* Psychischer Missbrauch, *T74.8* Sonstige Formen des Missbrauchs, *T74.9* Missbrauch von Personen, nicht näher bezeichnet, *1‑945.0* Diagnostik bei Verdacht auf Gefährdung von Kindeswohl und Kindergesundheit – ohne weitere Maßnahme, *1‑945.1* Diagnostik bei Verdacht auf Gefährdung von Kindeswohl und Kindergesundheit – mit Durchführung von mindestens einer spezifisch protokollierten Fallkonferenz

Je nach Misshandlungsform sind geschlechtsspezifische Unterschiede in den Häufigkeiten der Kodierungen zu erkennen (Abb. 2a). So wurden die Codes für sexuellen Missbrauch, psychischen Missbrauch und sonstige Formen des Missbrauchs über die letzten 5 Jahre bedeutend häufiger bei weiblichen als bei männlichen Personen kodiert. Die auffälligste Differenz in der prozentualen Häufigkeit besteht bei dem Code T74.2 sexueller Missbrauch: Knapp 85 % sind auf weibliche Personen zurückzuführen und lediglich 15 % auf männliche.

Abb. 2b zeigt die Verteilungen der Altersklassen der misshandlungsrelevanten Codes. Besonders prägnant zeichnen sich hier die Fälle der unter 1‑Jährigen ab, die bei den Diagnosecodes T74.0, T74.1 und T74.8 ca. ein Drittel der Fälle ausmachen. Ähnlich verhält es sich bei den verschlüsselten Prozeduren, bei denen ebenfalls ein beachtlicher Anteil der Fälle auf Säuglinge im Alter bis zu einem Jahr entfällt (1-945.0: 32,65 %, 1‑945.1: 47,73 %). Sexueller Missbrauch und psychischer Missbrauch werden mit 45,66 % und 37,62 % überwiegend bei Kindern und Jugendlichen im Alter von 10–15 Jahren erfasst.

### Misshandlungsrelevante Hauptdiagnosen.

Die Analyse der kodierten T74.x-Nebendiagnosen (*n* = 6156) und der dazu verschlüsselten Hauptdiagnosen (*n* = 3365) zeigt, dass im Zusammenhang mit Kindesmisshandlung bestimmte Diagnosegruppen häufiger als Hauptdiagnosen auftreten. Dies legt nahe, dass spezifische medizinische Zustände und Verletzungen in Kliniken und Krankenhäusern häufiger mit Misshandlung assoziiert werden. Die meisten Hauptdiagnosen sind dem ICD-10-Kapitel S00-T98 „Verletzungen, Vergiftungen und bestimmte andere Folgen äußerer Ursachen“ (25,05 %) zuzuordnen. Innerhalb dieses Kapitels zeigt sich eine deutliche Verbindung von Verletzungen des Kopfes mit Kindesmisshandlung (15,70 %). Die weiteren Hauptdiagnosegruppen im Zusammenhang mit Misshandlung weisen größtenteils eine unspezifische Verteilung auf, was aus geringen Prozentsätzen im Bereich von 0,5–2 % ersichtlich wird. Die weiteren Verteilungen der Hauptdiagnosen auf die Kapitel umfassen 14,15 % Faktoren, die zur Inanspruchnahme des Gesundheitswesens führen, 10,46 % psychische und Verhaltensstörungen und 5,08 % Zustände, die ihren Ursprung in der Perinatalperiode haben. Ähnlich verhält es sich bei den häufigsten Hauptdiagnosegruppen beim Prozess-Code zur Abklärung bei Verdacht auf Kindeswohlgefährdung (1-945.x.): Verletzungen, Vergiftungen und andere Folgen äußerer Ursachen (21,28 %), psychische und Verhaltensstörungen (14,24 %), Faktoren, die zur Inanspruchnahme des Gesundheitssystems führen (10,71 %) und Zustände, die ihren Ursprung in der Perinatalperiode haben (9,49 %).

### Zusammenhang T74.x und OPS 1-945.x.

**Abb. 3:**
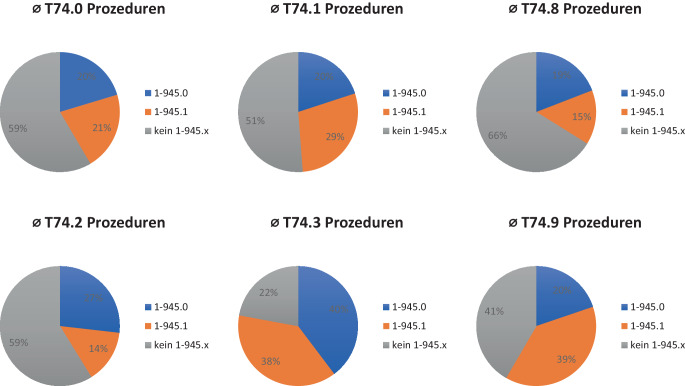
Zusammenhang zwischen vergebenen T74.x-Diagnosecodes und geleisteten 1‑945.x-Prozeduren. (Datenquelle: Institut für das Entgeltsystem im Krankenhaus (InEK); eigene Abbildung) *T74.x* „Missbrauch von Personen“ (Diagnosegruppe der Internationalen Klassifikation der Krankheiten – ICD-10), *1‑945.x* „Prozedur der Abklärung bei Verdacht auf Kindeswohlgefährdung“ (Diagnosegruppe des Operationen- und Prozedurenschlüssels – OPS), *T74.0* Imstichlassen oder Vernachlässigen, *T74.1* Körperlicher Missbrauch, *T74.2* Sexueller Missbrauch, *T74.3* Psychischer Missbrauch, *T74.8* Sonstige Formen des Missbrauchs, *T74.9* Missbrauch von Personen, nicht näher bezeichnet, *1‑945.0* Diagnostik bei Verdacht auf Gefährdung von Kindeswohl und Kindergesundheit – ohne weitere Maßnahme, *1‑945.1* Diagnostik bei Verdacht auf Gefährdung von Kindeswohl und Kindergesundheit – mit Durchführung von mindestens einer spezifisch protokollierten Fallkonferenz

Neben der Betrachtung der Ausprägungen der einzelnen Codes ist auch der Zusammenhang zwischen kodierten Diagnosen und Prozeduren relevant, da er Rückschlüsse auf die Zuverlässigkeit der Kodierung von Kindesmisshandlung ermöglicht. Die Auswertungen machen die oft fehlende Verknüpfung zwischen den Codes der ICD-10-Kodierungsgruppe T74.x und der Kodierung der Prozeduren OPS 1‑945.x deutlich: Insbesondere bei den als Nebendiagnose kodierten Misshandlungsformen „Vernachlässigen/Imstichlassen“, „Körperlicher Missbrauch“, „Sexueller Missbrauch“ und „Missbrauch von Personen, nicht näher bezeichnet“ werden bei über der Hälfte der Fälle (51–66 %) keine 1‑945.x-Codes abgerechnet (Abb. 3). Identisch verhält sich die Verknüpfung bei geleisteten Prozeduren 1‑945.x: Nur in 21 % der Fälle, bei denen 1‑945.0 abgerechnet wurde, erfolgte eine Kodierung eines T74.x-Codes; bei 1‑945.1 war dies bei 30 % der Fall.

## Diskussion

Diese Studie liefert erstmals eine systematische Auswertung der (teil-)stationären Daten zu Kindesmisshandlung und -vernachlässigung, die im G‑DRG-System in Kliniken und Krankenhäusern deutschlandweit dokumentiert wurden.

### Mangelnde Dokumentation von Kindesmisshandlung und -vernachlässigung in Kliniken.

Zur Bewertung der Größenordnung der verfügbaren Krankenhausdaten ist der Vergleich mit Daten aus dem Dunkelfeld interessant: Die Anzahl kodierter Kindesmisshandlungen steht in keinem Verhältnis zur Anzahl an Fällen, die aufgrund der Prävalenzen in der gesamtdeutschen Bevölkerungsstudie von Witt et al. [1] erwartet werden könnten. Krankenhausdaten aus den Jahren 2019 bis 2023 zeigen, dass jährlich durchschnittlich 549 von etwa 14,0 Mio. Kindern und Jugendlichen in Deutschland als Fälle von körperlicher Misshandlung gemäß ICD-10-GM kodiert wurden, was lediglich 0,004 % der Gesamtbevölkerung dieser Altersgruppe entspricht. Selbst unter Berücksichtigung der jährlich in Kliniken behandelten pädiatrischen Fälle – etwa 1,9 Mio. – liegt der Anteil mit 0,03 % nur geringfügig höher. Im Vergleich dazu gehen Witt et al. bei Kindern und Jugendlichen von einer Prävalenz von 12,4 % für leichten bis schweren körperlichen Missbrauch aus. Zwar ist ein solcher Vergleich von Daten aus dem Hell- und Dunkelfeld nur bedingt valide, da von Witt et al. Prävalenzdaten^4^ vorliegen, während die Kodierungen in Kliniken eher eine Inzidenzstatistik^5^ pro Jahr abbilden. Dennoch weisen die hohen Prävalenzen aus dem Dunkelfeld im Zusammenhang mit den niedrigen Inzidenzen der Kodierungen von Kindesmisshandlung auf erhebliche Herausforderungen in der Erkennung und Meldung von Kindesmisshandlung und eine unzureichende Erfassung hin. Die hohen Dunkelziffern deuten darauf hin, dass die Prävalenz von Kindesmisshandlung erheblich unterschätzt wird [20].

**Abb. 4:**
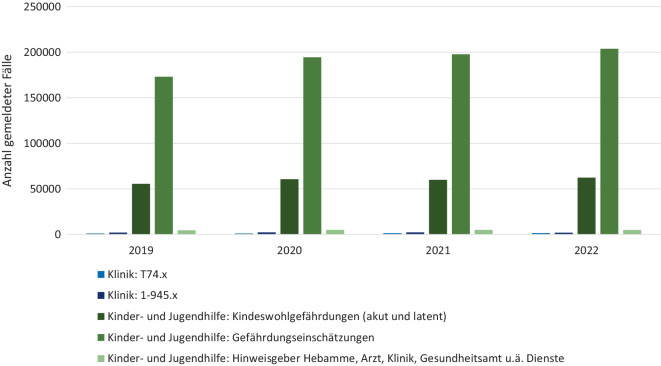
Kodierungen von Kindesmisshandlung in Kliniken und Anzahl Kindeswohlgefährdungen im Rahmen der Kinder- und Jugendhilfestatistik. (Datenquellen: Institut für das Entgeltsystem im Krankenhaus (InEK); Daten zu Kinder- und Jugendhilfestatistik entnommen aus: [21]; eigene Abbildung) *T74.x* „Missbrauch von Personen“ (Diagnosegruppe der Internationalen Klassifikation der Krankheiten – ICD-10), 1‑945.x „Prozedur der Abklärung bei Verdacht auf Kindeswohlgefährdung“ (Diagnosegruppe des Operationen- und Prozedurenschlüssels – OPS)

Eine unzureichende Dokumentation und Erfassung von Kindesmisshandlung im klinischen Setting wird zudem durch einen Vergleich mit Inzidenzraten aus anderen administrativen Datensätzen deutlich: Hier zeigt sich eine Diskrepanz zwischen der Anzahl kodierter Fälle (bei Verdacht auf) Kindesmisshandlung in Kliniken, an Jugendämter gemeldeter Gefährdungseinschätzungen durch Gesundheitspersonal und der Gesamtzahl von Jugendämtern erfasster Fälle bei (Abklärung bei Verdacht auf) Kindeswohlgefährdung (Abb. 4). Trotz der Schlüsselfunktion, die Ärzt*innen bei der Erkennung und Diagnostik von Kindesmisshandlung einnehmen [7], wurden 2021 gerade einmal 6 % der Gefährdungseinschätzungen im Rahmen der Kinder- und Jugendhilfe von Gesundheitspersonal angeregt [2]. Darüber hinaus zeigt sich in der Kinder- und Jugendhilfe seit 2012 langfristig ein deutlicher Anstieg der Fälle von Kindeswohlgefährdungen, während die Krankenhausdaten lediglich einen geringfügigen Anstieg verzeichnen.

Die Untererfassung der Kodierung von Kindesmisshandlung und -vernachlässigung wird auch bereits in nationaler und internationaler Forschung aufgezeigt [10–12]. Unter anderem untersuchten Garza et al. (2021) die Genauigkeit der Kodierung in Krankenhausdatenbanken mit dem Ergebnis, dass die Sensitivität der Diagnose von körperlichem Kindesmissbrauch bei stationären Patient*innen mit der Kodierung nach ICD-10-CM (Clinical Modification) gerade einmal bei etwa 55,6 % liegt (95 %-KI 41,4 % bis 69,1 %; [22]). Gründe für die Unterfassung wurden im Rahmen des TICANDAC-Projektes identifiziert: Unter anderem spielen Unsicherheiten bei der Identifizierung von Kindesmisshandlung, fehlendes Wissen über Kodierungssysteme, Angst vor Stigmatisierung der Eltern und Ressourcenmangel eine entscheidende Rolle. Darüber erweist sich die multiprofessionelle Zusammenarbeit bei Kinderschutzfällen als herausfordernd.^6^

Bestehende Literatur macht deutlich, dass der Mangel an validen Daten zur Kindesmisshandlung auch in anderen Versorgungsystemen im Hellfeld, wie beispielsweise bei Krankenkassen [23], der ambulanten Gesundheitsversorgung, Familiengerichten und der freien Kinder- und Jugendhilfe [10], ein enormes Problem darstellt.

Die insgesamt höheren und zunehmenden Fallzahlen der Kodierung des Operationen- und Prozedurenschlüssels 1‑945.x können auf verschiedene Aspekte zurückgeführt werden: Im Gegensatz zur Diagnosegruppe T74.x ist die Diagnosegruppe 1‑945.x seit 2018 für die Abrechnung von stationären Behandlungsfällen relevant, wodurch für Kliniken und Krankenhäuser ein Anreiz für die Kodierung besteht und erstmals die „massiven, vor allem personellen, zusätzlichen Aufwände, die in der medizinischen Kinderschutzarbeit entstehen“, berücksichtigt werden [24]. Zum anderen ist zu betonen, dass die Diagnosegruppe 1‑945.x die Möglichkeit bietet, bereits den Verdacht auf Kindeswohlgefährdung zu kodieren, wodurch auch Fälle dokumentiert werden (können), die nicht eindeutig sind. Ein Anstieg der Kodierungen könnte darüber hinaus insbesondere auch auf Bemühungen der Deutschen Gesellschaft für Kinderschutz in der Medizin (DGKiM) in Form von Fortbildungen, Zertifizierungen, Newslettern und Handlungsempfehlungen zurückzuführen sein. Aufgrund der oft fehlenden Verknüpfung von bestätigten T74.x-Fällen und OPS 1‑945.x und den dennoch geringen Fallzahlen besteht jedoch auch hinsichtlich der Verschlüsselung und Abrechnung der Diagnosegruppe 1‑945.x weiterhin Bedarf an Sensibilisierung und Aufklärung.

Der im Rahmen der Ergebnisse erkennbare Anstieg der Kodierung von Imstichlassen und Vernachlässigen im Jahr 2021 könnte möglicherweise auf eine Zunahme von Fällen von Vernachlässigung während des Corona-Lockdowns hinweisen: So berichtet eine Studie aus den Niederlanden beispielsweise bei vergleichender Betrachtung derselben Population vor und während des Lockdowns von einer Zunahme von Kindesvernachlässigung um das 3‑Fache [25]. Allerdings gestaltet sich die internationale Befundlage hier je nach Datenquelle unterschiedlich [26].

### Verhältnis Misshandlungsformen.

Die (teil-)stationären Daten zur Kodierung von körperlichem Missbrauch, sexuellem Missbrauch, Vernachlässigung und Imstichlassen und psychischem Missbrauch umfassen jeweils einen prozentualen Anteil von 45 %, 10 %, 33 % und 0,8 %, womit deutlich wird, dass körperlicher Missbrauch im stationären Setting am häufigsten kodiert wird. Auch der Blick auf die misshandlungsrelevanten Hauptdiagnosen zeigt die mit Kindesmisshandlung primär assoziierten körperlichen Verletzungen. Eine mögliche Erklärung hierfür ist, dass die Statistik durch den Fokus auf (teil-)stationäre Fälle vor allem Patient*innen mit Verletzungen nach akutem Gewalterleben abbildet. Darüber hinaus sind sichtbare Verletzungen potenziell eindeutiger zu erkennen und zu kodieren.^7^

Die genaue Betrachtung der Häufigkeit von Kodierungen der verschiedenen Misshandlungsformen der (teil-)stationären Daten ist dennoch wichtig, da diese sich von den aktuellsten repräsentativen Daten zu Kindesmisshandlung im Dunkelfeld in Deutschland unterscheiden: Während körperlicher Missbrauch innerhalb der (teil-)stationären Daten ca. 22 Mal häufiger als psychischer Missbrauch kodiert wird, weisen Prävalenzen aus dem Dunkelfeld darauf hin, dass moderater bis schwerer psychischer Missbrauch (6,5 %) und moderater bis schwerer körperlicher Missbrauch (6,6 %) nahezu gleich häufig auftreten [1]. Somit wird anhand der (teil-)stationären Daten deutlich, dass im klinischen Setting weniger offensichtliche Misshandlungsformen, wie beispielsweise psychischer Missbrauch, weniger dokumentiert werden. Die Schwierigkeiten bei der Identifizierung von psychischem Missbrauch und Vernachlässigung bestehen unter anderem darin, eindeutige Beweise zu erbringen, was sich auch im akademischen Diskurs widerspiegelt [27]. Diese Unsicherheiten bei weniger offensichtlichen Misshandlungsformen erfordern deshalb eine verstärkte Sensibilisierung und Unterstützung der Fachkräfte im Gesundheitswesen. Dass gezielte Fortbildungen, Sensibilisierung und strukturelle und konzeptionelle Neuorganisation der Kinderschutzarbeit in Kliniken effektiv sind, zeigt eine Untersuchung in einer Kinderklinik in Deutschland: Erkennbar wird nicht nur eine signifikant höhere Erfassung von (Verdachtsfällen von) Misshandlung, sondern auch eine Verschiebung der Häufigkeiten der Misshandlungsformen [13]. So werden nach interner Umstrukturierung mehr erhärtete Fälle von Vernachlässigung als von körperlichem Missbrauch identifiziert und auch sexueller Missbrauch wurde häufiger identifiziert, was eine Annäherung an die Verhältnisse im Dunkelfeld darstellt.^8^ Aufgrund der Ergebnisse gehen die Autor*innen von einer Verschiebung der Fälle aus dem Dunkelfeld ins Hellfeld aus.

### Häufigkeitsverteilungen Geschlecht und Alter.

Anhand der Daten ist zu erkennen, dass Jungen und Mädchen bei verschiedenen Misshandlungsformen unterschiedlich häufig erkannt werden, obwohl das allgemeine Risiko für Kindesmisshandlung außer bei sexuellem Missbrauch in Abhängigkeit vom Geschlecht nicht erhöht ist. In ihrer Metaanalyse berichten Stoltenborgh et al. (2011), dass die Unterschiede in den Prävalenzraten zu sexuellem Missbrauch bei Mädchen (18 %) und Jungen (7,6 %) statistisch signifikant sind [29]. Auch Jud et al. (2016) zeigen auf, dass Daten von Behörden und Erhebungen in der Bevölkerung übereinstimmend zu dem Ergebnis kommen, dass die Anzahl an Betroffenen von sexuellem Kindesmissbrauch bei Mädchen höher ist als bei Jungen [30]. Bei der Kodierung von sexuellem Missbrauch ist dementsprechend auch bei den erhobenen Daten in Kliniken die beobachtbare Diskrepanz von männlichen (15 %) und weiblichen Personen (85 %) am größten.

Körperliche Misshandlung hingegen wird eher mit männlichen Personen assoziiert. Internationale Prävalenzstudien und stationär erhobene Daten deuten darauf hin, dass männliche Personen häufiger von körperlicher Misshandlung betroffen sind. So zeigt beispielsweise das systematische Review von Moody et al. (2018), dass die Prävalenz von körperlicher Misshandlung im Dunkelfeld in Europa bei Jungen (27 %) deutlich höher ist als bei Mädchen (12 %; [31]). Auch im Rahmen des klinischen Settings zeigt sich bei Jungen eine erhöhte Prävalenz.^9^ Die Unterschiede in den Studien erweisen sich jedoch nicht als statistisch signifikant. Die auffallend hohe Anzahl an Säuglingen bei Fällen von körperlicher Misshandlung stimmt mit Ergebnissen aus nationalen und internationalen Studien überein [13, 16, 32]. Dies könnte darauf zurückzuführen sein, dass sehr junge Kinder mit Frakturen, die auf Missbrauch zurückzuführen sind, eher ins Krankenhaus eingeliefert werden als ältere Kinder [15]. Die These wird bei Betrachtung der Gesamtstichprobe gestärkt: Säuglinge unter einem Jahr machen die Hälfte (49 %) aller stationären Klinikaufenthalte aus.

### Limitationen.

Die vorliegende Analyse ist aufgrund der Verfügbarkeit der Daten auf den Zeitraum von 2019 bis 2023 beschränkt. Dadurch können in der Analyse eventuelle bedeutende Entwicklungen oder Trends in der Kodierung von Kindesmisshandlung vor dem Jahr 2019 nicht berücksichtigt werden.

Die geringen Fallzahlen zu Kindesmisshandlung können darüber hinaus zu stärkeren Schwankungen der prozentualen Angaben führen. Um dieser Limitation bestmöglich entgegenzuwirken, erfolgte jeweils die Berechnung der Durchschnittswerte der letzten 5 Jahre.

Durch den Fokus des InEK-Datenbrowsers auf (teil-)stationäre Fälle wurden bei den Analysen keine ambulanten Fälle berücksichtigt. Ambulante Patient*innen, bei denen in Kliniken oder Krankenhäusern eine Form von Kindesmisshandlung kodiert wird, sind in den Daten somit nicht enthalten.

Eine weitere Limitation besteht darin, dass die Daten keine Rückschlüsse darauf ermöglichen, ob es sich bei den Kodierungen der Jahre 2019–2023 jeweils um neue Fälle handelt oder bei einer Person im Laufe der Jahre mehrmals Kindesmisshandlung dokumentiert wurde. Die Daten bilden rein die Anzahl der jährlichen Fälle ab, bei denen die Codes vergeben wurden.

Darüber hinaus ist anzumerken, dass die Gegebenheiten in den Jahren der COVID-19-Pandemie potenziell Auswirkungen auf die Anzahl stationärer Aufenthalte hatten. Dennoch bleiben die prozentualen Verhältnisse der Erfassungen aussagekräftig, um mögliche Veränderungen im Vergleichszeitraum angemessen zu berücksichtigen.

## Fazit

Die Analyse verfügbarer Daten (teil-)stationärer Krankenhausaufenthalte macht die enorme Untererfassung aller Misshandlungsformen in Kliniken in Deutschland und damit verbunden die dringliche Notwendigkeit einer optimierten Datenerfassung deutlich. Obschon Daten zur Erfassung von Kindesmisshandlung aus dem Hellfeld verfügbar sind, zeigt sich eine erhebliche Diskrepanz zu den Prävalenzraten aus dem Dunkelfeld. Abgesehen von der gravierenden Untererfassung aller Misshandlungsformen, wird bei differenzierterer Betrachtung der Ergebnisse insbesondere das fehlende Bewusstsein für weniger offensichtlich erkennbare Misshandlungsformen ersichtlich.

Neben dem Bedarf der Sensibilisierung und Aufklärung der Fachkräfte in Kliniken und Krankenhäusern wird darüber hinaus auch die Notwendigkeit von Investitionen in politische Veränderungen deutlich: Insbesondere die mangelnde Erlösrelevanz in Fällen von Kindesmisshandlung ist zu diskutieren, aber auch strukturelle Rahmenbedingungen wie Personalressourcen müssen thematisiert werden.

Insgesamt unterstreicht die Analyse der kindesmisshandlungsrelevanten Codes die Notwendigkeit einer kontinuierlichen Qualitätsverbesserung der Dokumentation von Kindesmisshandlung in Kliniken, um die Überwachung von Kindesmisshandlung zu optimieren und gezielte und effektive Interventionen und Präventionsmaßnahmen für gefährdete Kinder und Jugendliche zu ermöglichen. Die Studie bietet hierfür erste Ansatzpunkte für eine verbesserte Datenerfassung und -dokumentation.

## Footnotes

1 https://elearning-childprotection.de/; zugegriffen am: 25.04.2024.

2 Da die Vergabe von T74.x-Codes als Hauptdiagnose in der Praxis unüblich ist, gibt es im InEK-Datenbrowser für 2019–2023 hierzu keine oder zu wenige Daten (erst ab 5 Fällen angezeigt).

3 In der amtlichen Übersetzung der ICD-10 wird „abuse“ als „Missbrauch“ übersetzt. Im deutschen Sprachgebrauch wird Missbrauch ausschließlich im Zusammenhang mit sexuellem Missbrauch verwendet. Ansonsten überwiegt die Verwendung des Begriffes „Misshandlung“.

4 Die Prävalenzdaten beschreiben den gesamten Anteil der Personen, die während ihrer Kindheit und Jugend von Kindesmisshandlung betroffen sind.

5 Die Inzidenz bezeichnet die Anzahl neu auftretender Fälle von Kindesmisshandlung in einer gegebenen Population während eines bestimmten Zeitraums.

6 Walter T, Korhonen L, van Agthoven G, Otterman G, Jud A (2024). Challenges to Reliable ICD-10 Coding of Child Maltreatment: A Qualitative Study of Healthcare Professionals in German and Swedisch Hospitals (**unveröffentlicht**).

7 Walter T, Korhonen L, Otterman G, van Agthoven G, Jud A (2024). Challenges to Reliable ICD-10 Coding of Child Maltreatment: A Qualitative Study of Healthcare Professionals in German and Swedisch Hospitals (**unveröffentlicht**).

8 Auch Daten aus Dunkelfeldstudien können nicht als verzerrungsfrei betrachtet werden. Limitationen von retrospektiven Dunkelfeldstudien sind unter anderem Verzerrungen durch den sogenannten Recall-Bias [1, 28].

9 Walter T, Korhonen L, Otterman G, van Agthoven G, Jud A (2024). Challenges to Reliable ICD-10 Coding of Child Maltreatment: A Qualitative Study of Healthcare Professionals in German and Swedisch Hospitals (**unveröffentlicht**).
